# ToxNav germline genetic testing and PROMinet digital mobile application toxicity monitoring: Results of a prospective single‐center clinical utility study—PRECISE study

**DOI:** 10.1002/cam4.2529

**Published:** 2019-09-04

**Authors:** Lennard Y. W. Lee, Thomas Starkey, Shivan Sivakumar, Susan Fotheringham, Guy Mozolowski, Vanessa Shearwood, Claire Palles, Philip Camilleri, David Church, Rachel Kerr, David Kerr

**Affiliations:** ^1^ Institute of Cancer and Genomic Sciences University of Birmingham Birmingham UK; ^2^ Department of Oncology University of Oxford Oxford UK; ^3^ Oxford Cancer Biomarkers Oxford Science Park Oxford UK; ^4^ Radcliffe Department of Medicine University of Oxford Oxford UK

**Keywords:** chemotherapy, Colorectal cancer, DPYD, ENOSF1, fluoropyrimidine, genetic testing, germline, toxicity, toxicity monitoring

## Abstract

**Introduction:**

In this study (PRECISE), we assess the clinical utility of a germline DNA sequencing‐based test (ToxNav) for mutations in *DPYD* and *ENOSF1* genes to alter clinician‐prescribed fluoropyrimidine doses and the use of a digital application (PROMinet) to record patient‐reported chemotherapy toxicity.

**Materials and methods:**

Adult patients with a histological diagnosis of colorectal cancer (CRC) who consented to fluoropyrimidine‐based chemotherapy were recruited prospectively and given a digital application to monitor and record associated toxicities. Patient samples were analyzed for 18 germline coding variants in *DPYD* and 1 *ENOSF1* variant.

**Results:**

Genetic testing was performed for 60 patients and identified one patient at increased risk of fluoropyrimidine‐based toxicities. Uptake of genetic testing was high and results were available on average 17 days from initial clinical encounter. Patient‐reported chemotherapy toxicity identified differences in 5‐fluorouracil vs capecitabine regime profiles and identified profiles associated with subsequent need for chemotherapy dose reduction and hospital admission.

**Discussion:**

The PRECISE clinical trial demonstrated that a germline DNA sequencing‐based test can provide clinically relevant information to alter clinicians' fluoropyrimidine prescription. The study also obtained high volume, high granularity patient‐reported toxicity data that might allow the improvement and personalization of chemotherapy management.

## Graphical abstract

In the PRECISE study, we assess the clinical utility of a germline DNA sequencing‐based test to alter clinician‐prescribed fluoropyrimidine doses and the use of a digital application to record patient‐reported chemotherapy toxicity. Genetic testing identified one patient at increased risk of fluoropyrimidine‐based toxicities and patient‐reported toxicity identified differences in chemotherapy regime profiles and association with subsequent need for chemotherapy dose reduction and hospital admission. The study demonstrated the clinical utility of a germline DNA sequencing‐based test for providing information to alter clinicians' fluoropyrimidine prescription and obtained high volume, high granularity patient‐reported toxicity data.

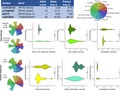

## 1 INTRODUCTION

5‐fluorouracil (5‐FU) and its oral prodrug capecitabine are the most commonly prescribed chemotherapeutic agents for treating colorectal cancer (CRC) and feature prominently in many chemotherapy regimens. A significant proportion (20%‐30%) of patients treated with these drugs develop severe side effects,^1^, ^2^, ^3^, ^4^ usually observed in the first cycle of treatment, which may result from inborn deficiencies of enzymes or drug transporters used by the body for drug break down and deactivation.^2^, ^5^, ^6^, ^7^

Severe toxicity and toxic deaths among patients receiving chemotherapy arise from two main mechanisms. Firstly, the patient has an unrecognized enzyme deficiency and is prescribed a “standard” chemotherapy dose, resulting in severe side effects developing during the first chemotherapy cycle.^8^, ^9^, ^10^, ^11^ Secondly, severe toxicity may occur in patients who have under/unreported or unrecognized chemotherapy toxicities.^12^ To prevent severe toxicity, it is important to develop new methods to recognize pharmacogenetic differences in drug metabolism that predispose a patient to suffer severe toxicities. There is also a need to develop improved methods to accurately record patient toxicity.

Dihydropyrimidine dehydrogenase (*DPYD*) plays the dominant role in 5‐FU and capecitabine degradation, and reduced enzyme activity leads to toxicity resulting from toxic metabolite accumulation.^13^, ^14^ Large‐scale meta‐analyses have highlighted the importance of *DPYD* germline polymorphisms in causing toxicities in CRC clinical studies. Toxicity‐associated alleles are rare and seen in between 1.7% and 3.1% of the population.^13^, ^15^ To assess 5‐FU and capecitabine toxicity‐associated alleles, a 19 single nucleotide polymorphism (SNP) panel consisting of germline polymorphisms in *DPYD* and *ENOSF1* was determined and diagnostic accuracy was evaluated in 888 patients from the QUASAR 2 clinical trial.^16^ In brief, this study built on genome‐wide association studies and meta‐analyses performed using data available from a number of studies, including QUASAR 2, where genetic markers of toxicity resulting from fluoropyrimidine‐based therapies in the *DPYD* and *ENOSF1* genes were identified and assessed for their clinical utility.^15^, ^17^

The 19 germline genetic variants assessed for predicting fluoropyrimidine‐related toxicities (18 SNPs in *DPYD* and 1 SNP in *ENOSF1*) comprise the ToxNav test, a test which demonstrated superior performance when compared with other diagnostic tests for assessing fluoropyrimidine‐related toxicities currently available in the United Kingdom.^16^ Following on from the clinical trial, the first objective of the PRECISE pilot study was to assess the feasibility of ToxNav test delivery within 2 weeks of initial patient consultation and whether clinicians will use the ToxNav test to change, a priori, previously prescribed fluoropyrimidine‐based chemotherapy doses.

Poor chemotherapy toxicity recognition is a problem recognized by clinicians. Some patients use a paper diary; however, the range and depth of information recorded are highly variable and subjective to personal experience.^18^, ^19^, ^20^, ^21^ Therefore, the utility of a digital mobile application (app), PROMinet, was assessed as the second objective. The app allows the prompted, daily self‐reporting of toxicity data as the patient proceeds through chemotherapy. It is hypothesized that this data will improve the breadth and depth of chemotherapy toxicity recording, identify patients at risk of severe side effects, and increase clinical awareness of needing formal dose reductions.

## 2 MATERIALS AND METHODS

### 2.1 Clinical trial

Ethical approval was obtained from the Oxford Ethics Committee to recruit patients into the PRECISE trial (REC reference: 16/LO/0915). The trial was a cohort observation clinical utility study. Patients were included if they had a histological diagnosis of CRC, age >18 years, and were to be prescribed fluoropyrimidine‐based chemotherapy. Exclusion criteria included patients previously treated with 5‐FU‐based chemotherapy, those with known contraindications to 5‐FU, pregnancy or breast‐feeding, and an inability to use a mobile digital device.

### 2.2 ToxNav genetic testing

The ToxNav test is a sequencing‐based analysis of 18 genetic variants in *DPYD* and one in *ENOSF1*. Genetic markers were identified through the use of microarray genotyping and included if they met one of the following criteria: 1. Minor allele frequency (MAF) <1%, identified in patients with DPYD deficiency (three of these markers were also associated with toxicity at *P* < .05). 2. MAF >1% and associated with global capecitabine‐related toxicity with an odds ratio >1.5 at pathway level significance and associated with an individual toxicity at genome‐wide significance. This approach identified that these DPYD variants had a high sensitivity and specificity when toxicity‐induced death or grade 4 hematological toxicities are the outcome of interest (100% sensitivity, 98% specificity, negative predictive value (NPV) 1.0, positive predictive value (PPV) 0.1 (death); 75% sensitivity, 98% specificity, NPV 1, PPV 0.14 (hematological toxicities).^16^

Written consent was obtained from each patient for genetic testing. Five milliliter of blood was taken into an EDTA bottle, and anonymized with a unique identifier code. DNA was extracted from whole blood with samples processed within 96 hours of blood collection. Twenty‐eight PCRs were performed to provide full coverage of *DPYD* (23 exons) and the variant region of *ENOSF1*. Interpretation of these data was performed according to a predesigned algorithm, ColoTox software v8.0.0.2, and a risk report result based on the known penetrance of the variance to chemotherapy toxicity, made available to the treating physician designating them into one of four categories (Table 1, Supplementary Figure S1).

**Table 1:** Risk classification criteria for assigning a risk classification to patients based on the genetic sequencing results from the ToxNav test

Risk classification	Risk classification criteria
Critical risk	Patient carried two deficiency alleles (homozygous for a single DPYD deficiency allele or heterozygous for two DPYD deficiency alleles).
High risk	Patient carried one copy of a DPYD deficiency allele.
Standard risk	Patient carried no copies of DPYD deficiency alleles or HFS‐associated alleles.
Standard risk with high risk of HFS	Patient carried no copies of DPYD deficiency alleles but one or more alleles associated with increased risk of HFS.

### 2.3 Digital mobile application assessment

Digital mobile application (app)‐based monitoring of toxicities was performed through the use of a mobile tablet that was given to the patient or through an app downloaded to their mobile device (PROMinet), developed by *Oxford Medical Intelligence*. The app functions through the prompted, daily questionnaire of toxicity data as the patient proceeds through chemotherapy, and patients were given training on how to use the app before starting chemotherapy.

### 2.4 Statistics and toxicity data analyses

Toxicity data were analyzed within the R statistical package (version 3.5.2). App responses were averaged across treatment for each patient to give average responses for each week and across the 12‐week monitoring period.

## 3 RESULTS

### 3.1 Patient characteristics

Sixty patients were recruited into the PRECISE study. The median age of patients in the study was 63 years (stdev 12.5 years, range 31‐77 years). The majority of patients had stage III CRC and were prescribed CAPOX chemotherapy (75%), though FOLFOX (16.7%), single‐agent capecitabine (6.7%), and FOLFIRI (1.7%) were also administered (Table 2).

**Table 2:** Baseline characteristics of patient and chemotherapy indication and regime given

	No. (%)
Sex	
Male	32 (53.3)
Female	28 (46.7)
Age	
Median/ Std (y)	63/13.0
Tumor location	
Small bowel	1 (1.7)
Cecum	4 (6.7)
Ascending Colon	4 (6.7)
Hepatic Flexure	3 (5.0)
Transverse Colon	4 (6.7)
Splenic Flexure	4 (6.7)
Descending Colon	4 (6.7)
Rectosigmoid	26 (43.3)
Rectum	10 (16.7)
Microsatellite	
Microsatellite stable	48 (80.0)
Microsatellite instability	2 (3.3)
Not available	10 (16.7)
T‐stage	
pt1	2 (3.3)
pt2	3 (5.0)
pt3	26 (43.3)
pt4	19 (31.7)
Not available	10 (16.7)
N‐stage	
n0	12 (20.0)
n1	24 (40.0)
n2	14 (23.3)
Not available	10 (16.7)
Chemotherapy indication	
Adjuvant	41 (68.3)
Metastatic	18 (30.0)
Neoadjuvant	1 (1.7)
Chemotherapy regime	
Single‐agent CAP	4 (6.7)
CAPOX	45 (75.0)
FOLFOX	10 (16.7)
FOLFIRI	1 (1.7)

### 3.2 Genotyping data

ToxNav testing was performed on 59/60 patients in the trial. The test was not performed in one patient where the blood specimen was not received within the required 96 hours sample processing window. Results were available at a median of 17 days following the initial clinic appointment (stdev 8.1 days). However, for the last 10 patients tested, results were available at a median of 12.5 days (stdev 5.4 days).

From the test result, a risk classification was designated (Table 1). No patients were identified with a critical risk variant, one patient was identified with high‐risk variant A551T (*DPYD* Exon 13). Variants were frequently found in the *ENOSF1* (rs2612091) and *DPYD* intronic regions, rs7548189 and rs12132152 (Table 3, Supplementary Table S1).^15^ Positive results in rs72549303 were initially identified in three patients, but subsequently not validated.

**Table 3:** Germline variants analyzed in the ToxNav test and frequencies observed in the PRECISE clinical cohort

ToxNav ID	Gene	Variant	Allele count	Allele freq %	Patient freq %
Variant3	DPYD Intronic	rs12132152	4	3.39	6.78
Variant4	DPYD Intronic	rs7548189	15	12.71	23.73
Variant5	DPYD Exon 13	A551T	1	0.85	1.69
Variant6	TYMS/ENOSF1	rs2612091	54	45.76	66.10

### 3.3 Chemotherapy dose guided by ToxNav^TM^ assay

One patient received a ToxNav^TM^ test that suggested predisposition to a high risk of 5‐FU‐based chemotherapy toxicity. This patient had the initial dose reduced to 80% and subsequently experienced minimal/mild (Common Terminology Criteria for Adverse Events‐ CTCAE 0/1) side effects. Patients predicted to be at high risk of hand‐foot syndrome (HFS) were not dose‐reduced, but encouraged to use intensive moisturizing regimes and avoid astringent detergents.

During the study evaluation stage, outside of the PRECISE trial, two patients with upper GI cancer who had received one cycle of 5‐FU/cisplatin suffered severe (grade 4) neutropenia, sepsis, and subsequently died from their toxicities. These patients were genotyped retrospectively and found to have rs115232898 (high‐risk) and rs55886062 (critical risk) variants, respectively.

### 3.4 Chemotherapy toxicities—clinician recorded

About 58 of 60 patients started their chemotherapy regime, one patient's CRC progressed rapidly precluding chemotherapy and one patient withdrew consent. Of these, 52 patients had complete clinician‐reported toxicity data. Clinician‐graded toxicities identified that 55.8% (29/52) experienced minimal/mild side effects (CTCAE 0/1), 32.7% (17/52) experienced moderate side effects (CTCAE 2), and six patients had severe side effects (CTCAE 3) that required hospitalization due to colitis and/or diarrhea. About 38.5% of patients had their initially prescribed chemotherapy regime changed (20/52): four had their chemotherapy stopped, 11 had their chemotherapy dose‐reduced (due to gastrointestinal side effects, n = 6; HFS, n = 2; cardiac events, n = 4; oxaliplatin‐related toxicity, n = 4), five started a new chemotherapy regime due to toxicity or disease progression. Patients who received infusional 5‐FU were significantly less likely to experience moderate/severe side effects (CTCAE 3/4) (Chi‐squared *P* = .034).

### 3.5 Digital mobile application toxicity monitoring

Of 60 patients, 53 were recruited for PRECISE participated in the PROMinet app trial. Of these, 30 patients were provided with a tablet for recording toxicity severity responses and 23 patients downloaded the app onto their own mobile device.

**Figure 1:**
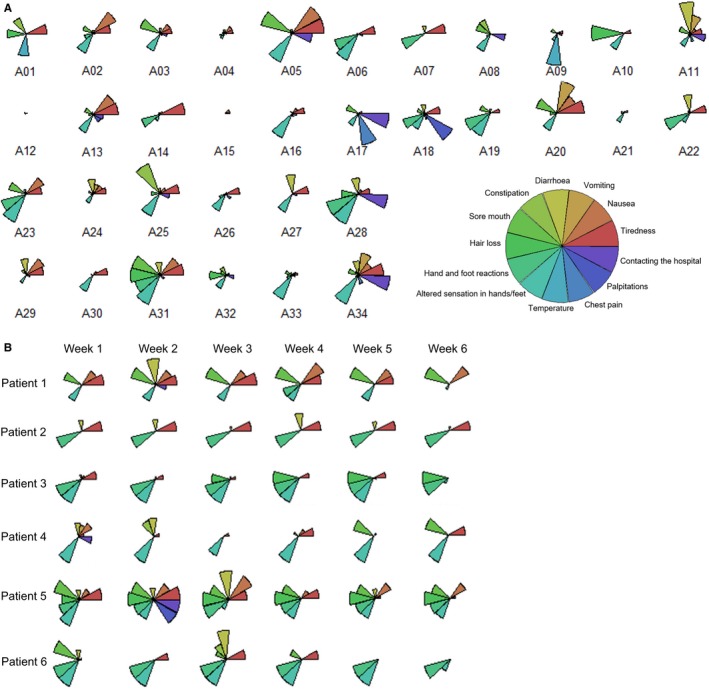
A, Individual patient chemotherapy toxicity profiles generated using app responses over the 84 day period. The star plot size for each toxicity is relative to the range of scores for each individual toxicity. B, Illustrative toxicity profiles demonstrate that profiles for each patient are consistent and reproducible across time

Patients using their own devices were significantly more likely to record app responses (87.0% vs 46.7%, *P* < .005). App responses were recorded for 34 patients, giving an uptake of 64.2%. A mean of 758 data points was collected for each patient, consisting of scores for 13 toxicity variables. The app response frequency recorded for each patient greatly varied, ranging from 10.6% to 97.6%, giving an average of 68.6% of days while on chemotherapy. Toxicity profiles for patients across the course of their chemotherapy regime were generated (Figure 1A). Toxicity profiles for each patient were distinct, but reproducible across chemotherapy cycles (Figure 1B).

### 3.6 Correlation between digital mobile application toxicity scores and clinician score

**Figure 2:**
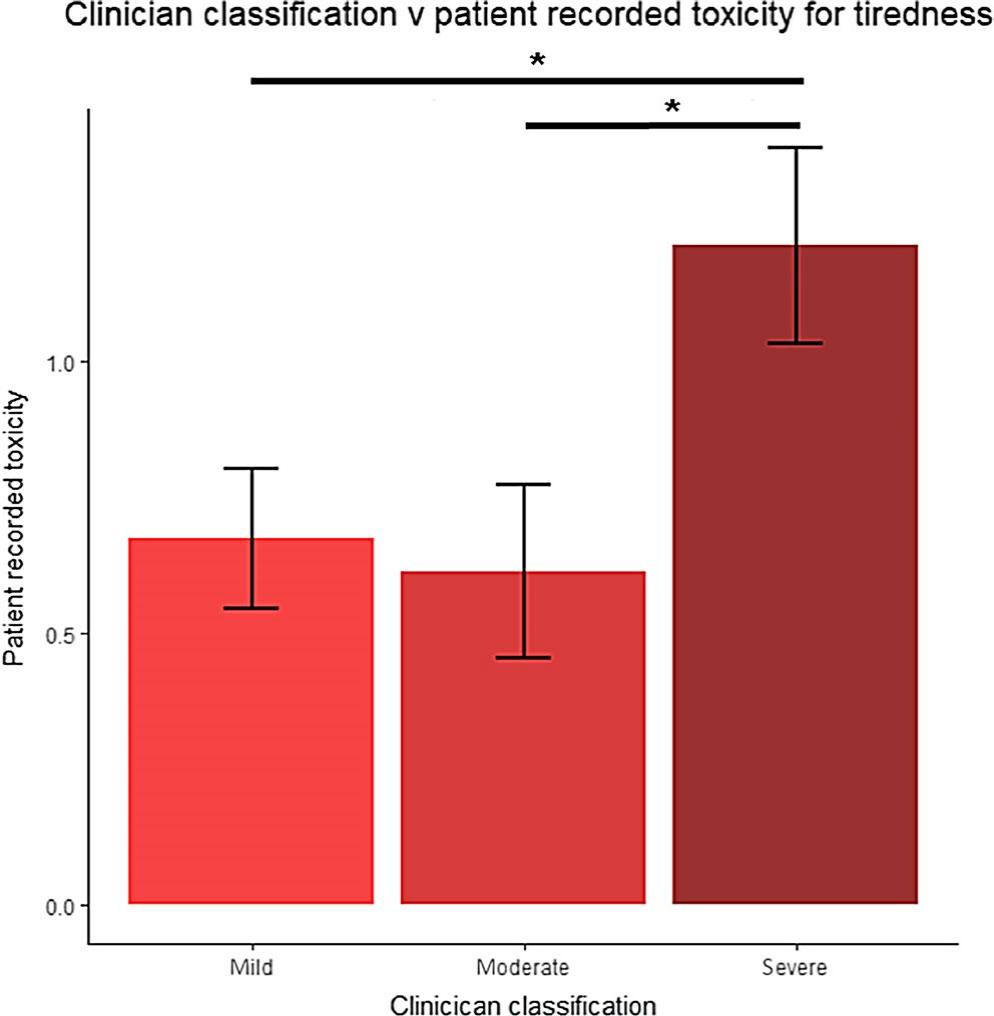
Digital mobile application patient recorded toxicity scores for “tiredness” during week 2 of chemotherapy. Patient recorded toxicity scores were significantly higher during week 2 of chemotherapy for patients classified as “severe” by clinicians compared to those classified as “mild” (1.215 vs 0.676, *P* = 0.048) or “moderate” (1.215 vs 0.615, *P* = 0.039)

Patient toxicity is determined by the clinician at each clinical encounter. As patients also recorded their own toxicities using the app, we sought to understand if these toxicity measures were correlated. We found that of the 13 app‐measured parameters, “tiredness” was significantly linked to clinician‐graded toxicity (clinician‐reported “severe” vs “moderate” or “mild,” *P* < .05 for both, Figure 2). Trends were also observed for incidence of “hand‐foot reactions,” “nausea,” and “sore mouth” whereby higher incidences were reported for patients where a “severe” clinician‐graded toxicity was given in comparison to patients where clinician‐graded toxicity was given as “moderate” or “mild,” though these were not statistically significant.

### 3.7 CAPOX vs 5‐FU chemotherapy

**Figure 3:**
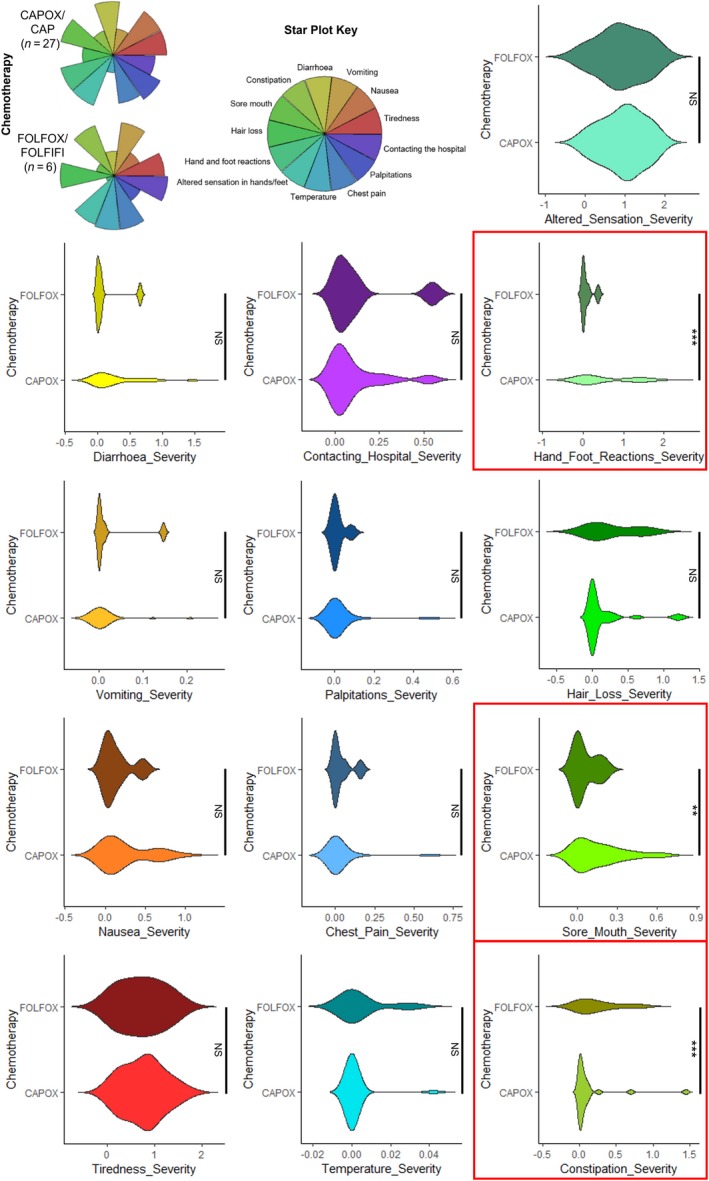
Toxicity profiles for patients receiving oral capecitabine vs intravenous 5‐fluorouracil chemotherapy regimens. Changes to hand and foot reactions and sore mouth were significantly more severe for patients treated with CAPOX/CAP whereas constipation was less severe. ***P < .001, **P < .01, *P < .05, NS = not significant

App responses for 13 symptom toxicities were analyzed for patients receiving either CAPOX/single‐agent CAP (n = 27), or FOLFOX/FOLFIRI (n = 6). In patients receiving CAPOX/CAP, the incidence of HFS (0.539 vs 0.082, *P* < .001) and sore mouth (0.163 vs 0.056, *P* < .01) was significantly higher. Patients receiving FOLFOX/FOLFIRI experienced a higher incidence of constipation (0.118 vs 0.245, *P* < .001) (Figure 3). Higher incidences of nausea (0.251 vs 0.133) and diarrhea (0.274 vs 0.126) were also observed for CAPOX/CAP‐treated patients, whereas the incidence of hair loss (0.154 vs 0.270) was higher in patients treated with FOLFOX/FOLFIRI, though these trends were not statistically significant.

### 3.8 Digital mobile application early predictors of need for dose reduction

Several patients had their starting chemotherapy dose reduced during subsequent cycles. We therefore sought to identify within app data for week 1 if there were potential factors that might identify patients where a subsequential chemotherapy dose reduction would be required. A linear regression model was utilized, and in patients who had subsequential chemotherapy dose reduction, severity of three toxicities significantly differed. “Hand‐foot reaction” was inversely correlated with subsequent need for dose reduction (*P* = .002), whereas “vomiting” (*P* = .021) and “diarrhea” (*P* = .024) were higher during week 1. The inverse relationship with HFS is surprising, but might suggest a non‐cell cycle effect as HFS is caused by perivascular lymphocytic and eosinophilic infiltrates.^22^

### 3.9 ToxNav and digital mobile application recording for hand‐foot syndrome

The ToxNav test also identifies patients at potential risk of developing “hand‐foot reactions/syndrome” (HFS). To assess utility, app responses for “hand‐foot reactions/syndrome” were compared to the ToxNav HFS risk score.

Of the patients who provided app responses, 22/34 were classified as “high‐risk HFS” and 12/34 as “low‐risk HFS” by the ToxNav test. For patients classified as “high‐risk HFS,” there was a trend for app‐recorded HFS severity to be higher during week 1 (0.209 vs 0.118) and week 2 (0.346 vs 0.167) of the app monitoring period, though this did not reach statistical significance (*P* = .392, *P* = .404, respectively). HFS was identified by treating physicians for three patients, of which one was identified as “high‐risk HFS” by the ToxNav test.

### 3.10 Hospital admissions and digital mobile application toxicities

**Figure 4:**
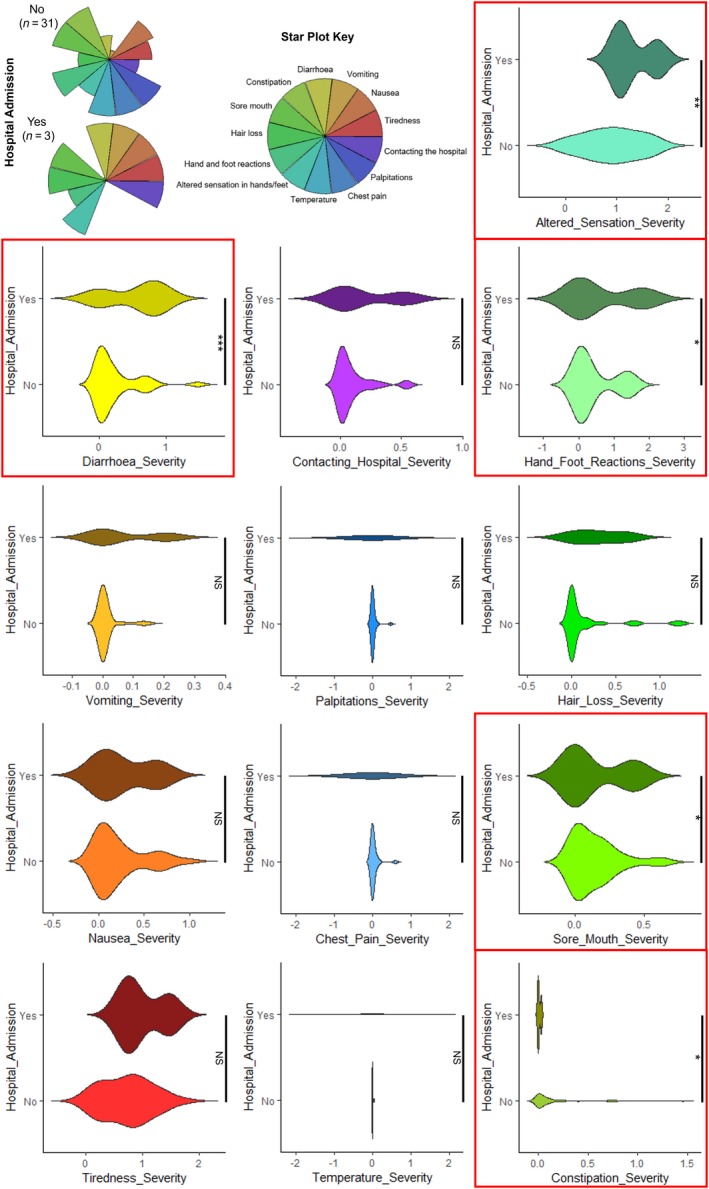
Toxicity profiles for patients that had severe toxicities requiring a hospital admission vs. those that did not. Diarrhoea and altered sensations in hands and feet were significantly more severe in patients admitted to hospital whereas constipation and sore mouth were less severe. ***P < .001, **P < .01, *P < .05, NS = not significant

Patients with severe toxicities (CTCAE 3/4) may require acute hospital admission. Three patients recruited into the PRECISE study, who participated in the app trial, were admitted to hospital during the study monitoring period. These patients received CAPOX chemotherapy and were hospital‐admitted during the first three cycles. We therefore sought to establish if the app toxicity data could better identify toxicity profiles of these patients by comparing app toxicity responses from patients who required hospitalization with those who did not. The spectra of toxicity were different between these groups and severity of “diarrhea” and “altered hand‐foot” was significantly elevated in patients requiring hospitalization, with lower scores for “constipation” and “sore mouth” (Figure 4).

## 4 DISCUSSION

A small proportion of the population carry rare genetic variants that likely account for the toxicity experienced in patients undergoing chemotherapy. Our understanding of the germline genetic determinants of 5‐FU‐based chemotherapy toxicity has advanced markedly and a significant proportion of this risk can be explained by *DPYD* and *ENOSF1* variants. By empowering clinicians with knowledge of their patient's genetic susceptibility, and enabling them to combine this with their clinical judgment, we propose this might reduce chemotherapy toxicities.

To date, the use of germline pharmacogenomic biomarkers has had a limited effect on clinical practice. Mutation profiling of the *TPMT* gene for patients receiving azathioprine and testing for HLA‐B*1502 for Asian patients being prescribed carbamazepine^23^ have entered clinical practice. In cancer research, there is no paucity of pharmacogenomic studies that have identified gene polymorphisms on chemotherapy toxicity; however, few have been assessed in routine clinical care.

Through the increasing availability of germline genetic sequencing tests, we predict that more clinically important information will become available to clinicians. Challenges will need to be surmounted to integrate germline variants associated with prognosis, disease response, or treatment toxicities and combine it with existing tests to maximize patient outcomes. The first aim of this clinical utility study was to determine whether a germline DNA sequencing‐based test, ToxNav, can provide clinically relevant information to assist and/or affect treatment decision‐making in patients receiving 5‐FU‐based chemotherapy. This study demonstrates that this is indeed achievable in the current UK oncology clinical practice and provides meaningful information in a timely manner.

Limitations to this pharmacogenomic aspect of the PRECISE study should be acknowledged. Firstly, germline DNA variants were identified from large cohorts of the UK‐based CRC patients. While it is reasonable to expect that they would predispose patients to fluoropyrimidine toxicity in other cancer or ethnicity settings, this requires validation. Secondly, this study does not claim to make any assertions of the efficacy of the ToxNav test in reducing overall patient toxicities and improving patient outcomes. Germline toxicity‐associated alleles are present in such a low frequency in the population (1.7%‐3.1%), that a much larger validation study would be required to assess ToxNav test efficacy and this study is currently recruiting. However, with regard to patients genotyped in this study, one patient at high risk of toxicity was dose‐reduced ab initio and tolerated chemotherapy without problems, whereas retrospective testing of two patients admitted with grade 4 neutropenia/sepsis were correctly identified as high risk. It is reasonable to speculate that if the test was available to these individuals they could have avoided severe fluoropyrimidine toxicity.

We believe this trial is important because it attempts to address a major issue which is unresolved in the field of pharmacogenomic research. Namely, that chemotherapy toxicity is a challenging phenotype to identify and quantify. Toxicity is subjective, a continuous variable, varies each day across a 2‐3 week chemotherapy cycle, entirely dependent on physician/patient recorded outcomes, and with no surrogate quantifiable biomarkers. Therefore in addition to assessing the pharmacogenetic test, we also utilized a novel mobile application (PROMinet) to collect patient feedback for 13 different chemotherapy‐associated toxicities. This mobile application addresses many of the limitations of identifying and quantifying chemotherapy toxicities. Our exploratory analyses suggest that the app could discriminate differences in chemotherapy toxicity profiles between patients receiving oral capecitabine and intravenous 5‐FU. We identified differing toxicity profiles for patients who subsequently required either a dose reduction or hospital admission. We anticipate the app will fit well in conjunction with existing clinical care and provide cost‐effective real‐time data, which may be used to reduce the likelihood of hospital admission by reducing chemotherapy dose and/or managing toxicities.

However, limitations to the app trial should be appreciated. The patient app response rate widely varied, resulting in differences in the quantity and quality of results obtained over the 84 days monitoring period. Furthermore, larger validation cohorts would be required to determine and demonstrate the accuracy and reliability of the PROMinet app (or equivalent) to identify patients who are likely to be admitted to hospital and/or have subsequential chemotherapy dose reduction.

Nevertheless, we demonstrate that the app improves the breadth and depth of toxicity data available. Future app development will enable it to give patient advice on managing individual side effects, and will in addition, alert clinicians to patients developing high‐grade toxicity that need to be contacted, and identify those who might benefit from preemptive contact.

In summary, the accurate pharmacogenomic prediction and monitoring of severe toxicity and toxic deaths among chemotherapy‐receiving patients have the potential to reduce morbidity and mortality. In this clinical utility study, we demonstrate that a genomic ToxNav test with concurrent monitoring using the PROMinet app provides potentially useful information to treating physicians and warrants further larger scale studies.

## CONFLICT OF INTEREST

Oxford Cancer Biomarkers Limited (OxfordBio) developed the ToxNav^TM^ germline DNA sequencing‐based test. Susan Fotheringham and Guy Mozolowski are employees of OxfordBio and David Kerr is a company director.

## AUTHOR CONTRIBUTIONS

LL‐ study concept and design, acquisition of data, analysis, interpretation of data, drafting of the manuscript, statistical analysis. TS‐ analysis, interpretation of data, drafting of the manuscript, statistical analysis. SS‐ patient recruitment, critical revision of the manuscript. SF‐ study supervision, critical review of the manuscript. GM‐ study supervision. VS‐ patient recruitment. CP‐ analysis, interpretation of data, statistical analysis, critical revision of the manuscript. PC‐ study concept and design, acquisition of data, interpretation of data, statistical analysis. DC‐ patient recruitment, critical revision of the manuscript. RK‐ patient recruitment, acquisition of data, study supervision, and obtained funding. DK‐ study concept and design, acquisition of data, interpretation of data, study supervision, and critical revision of the manuscript.

## References

[1] Grothey A , Sobrero AF , Shields AF , et al. Duration of adjuvant chemotherapy for stage III colon cancer. N Engl J Med. 2018;378:1177‐1188. 29590544 10.1056/NEJMoa1713709 PMC6426127

[2] Froehlich TK , Amstutz U , Aebi S , Joerger M , Largiadèr CR . Clinical importance of risk variants in the dihydropyrimidine dehydrogenase gene for the prediction of early‐onset fluoropyrimidine toxicity. Int J Cancer. 2014;136:n/a‐n/a. 10.1002/ijc.29025 24923815

[3] Meulendijks D , Henricks LM , Sonke GS , et al. Clinical relevance of DPYD variants c.1679T>G, c.1236G>A/HapB3, and c.1601G>A as predictors of severe fluoropyrimidine‐associated toxicity: a systematic review and meta‐analysis of individual patient data. Lancet Oncol. 2015;16:1639‐1650. 26603945 10.1016/S1470-2045(15)00286-7

[4] Lee AM , Shi Q , Pavey E , et al. DPYD variants as predictors of 5‐fluorouracil toxicity in adjuvant colon cancer treatment (NCCTG N0147). J Natl Cancer Inst. 2014;106. 10.1093/jnci/dju298 PMC4271081 25381393

[5] Amstutz U , Henricks LM , Offer SM , et al. Clinical pharmacogenetics implementation consortium (CPIC) guideline for dihydropyrimidine dehydrogenase genotype and fluoropyrimidine dosing: 2017 update. Clin Pharmacol Ther. 2018;103:210‐216. 29152729 10.1002/cpt.911 PMC5760397

[6] Caudle KE , Thorn CF , Klein TE , et al. Clinical Pharmacogenetics Implementation Consortium guidelines for dihydropyrimidine dehydrogenase genotype and fluoropyrimidine dosing. Clin Pharmacol Ther. 2013;94:640‐645. 23988873 10.1038/clpt.2013.172 PMC3831181

[7] Iveson TJ , Kerr RS , Saunders MP , et al. 3 versus 6 months of adjuvant oxaliplatin‐fluoropyrimidine combination therapy for colorectal cancer (SCOT): an international, randomised, phase 3, non‐inferiority trial. Lancet Oncol. 2018;19:562‐578. 29611518 10.1016/S1470-2045(18)30093-7 PMC5883334

[8] Feron O , Abalo R , Nurgali K , Jagoe RT . Editorial: adverse effects of cancer chemotherapy: anything new to improve tolerance and reduce sequelae? Front. Pharmacol. 2018;1:245 Available from: http://www.frontiersin.org. 10.3389/fphar.2018.00245 PMC5874321 29623040

[9] Livshits Z , Rao RB , Smith SW . An approach to chemotherapy‐associated toxicity. Emerg Med Clin North Am. 2014;32:167‐203. 24275174 10.1016/j.emc.2013.09.002

[10] Gustavsson B , Carlsson G , Machover D , et al. A review of the evolution of systemic chemotherapy in the management of colorectal cancer. Clin Colorectal Cancer. 2015;14:1‐10. 25579803 10.1016/j.clcc.2014.11.002

[11] Ruzzo A , Graziano F , Galli F , et al. Dihydropyrimidine dehydrogenase pharmacogenetics for predicting fluoropyrimidine‐related toxicity in the randomised, phase III adjuvant TOSCA trial in high‐risk colon cancer patients. Br J Cancer. 2017;117(9):1269‐1277. 29065426 10.1038/bjc.2017.289 PMC5709672

[12] Basch E , Deal AM , Kris MG , et al. Symptom monitoring with patient‐reported outcomes during routine cancer treatment: a randomized controlled trial. J Clin Oncol. 2016;34:557‐565. 26644527 10.1200/JCO.2015.63.0830 PMC4872028

[13] Latchman J , Guastella AM , Tofthagen C . 5‐fluorouracil toxicity and dihydropyrimidine dehydrogenase enzyme: implications for practice case study HHS public access. Clin J Oncol Nurs. 2014;18:581‐585. 25253112 10.1188/14.CJON.581-585 PMC5469441

[14] Chung T , Na J , Kim Y‐I , et al. dihydropyrimidine dehydrogenase is a prognostic marker for mesenchymal stem cell‐mediated cytosine deaminase gene and 5‐fluorocytosine prodrug therapy for the treatment of recurrent gliomas. Theranostics. 2016;6:1477‐1490. 27446484 10.7150/thno.14158 PMC4955049

[15] Rosmarin D , Palles C , Church D , et al. Genetic markers of toxicity from capecitabine and other fluorouracil‐based regimens: investigation in the QUASAR2 study, systematic review, and meta‐analysis. J Clin Oncol. 2014;32:1031‐1039. 24590654 10.1200/JCO.2013.51.1857 PMC4879695

[16] Palles C , Fotheringham S , Chegwidden L , et al. P‐213An evaluation of the clinical utility of a panel of variants in DPYD and ENOSF1 for predicting common capecitabine related toxicities. Ann Oncol. 2018;29. 10.3390/cancers13071497 PMC8037940 33805100

[17] Rosmarin D , Palles C , Pagnamenta A , et al. A candidate gene study of capecitabine‐related toxicity in colorectal cancer identifies new toxicity variants at DPYD and a putative role for ENOSF1 rather than TYMS. Gut. 2015;64:111‐120. 24647007 10.1136/gutjnl-2013-306571 PMC4283622

[18] Richardson A , Ream E . The experience of fatigue and other symptoms in patients receiving chemotherapy. Eur J Cancer Care (Engl). 1996;5:24‐30. 9117040 10.1111/j.1365-2354.1996.tb00248.x

[19] Rambach L , Bertaut A , Vincent J , et al. Prognostic value of chemotherapy‐induced hematological toxicity in metastatic colorectal cancer patients. World J Gastroenterol. 2014;20:1565. 24587632 10.3748/wjg.v20.i6.1565 PMC3925865

[20] Pearce A , Haas M , Viney R , et al. Incidence and severity of self‐reported chemotherapy side effects in routine care: a prospective cohort study. PLoS ONE. 2017;12:e0184360. 29016607 10.1371/journal.pone.0184360 PMC5634543

[21] Loree JM , Sha A , Soleimani M , et al. Survival impact of CAPOX versus FOLFOX in the adjuvant treatment of stage III colon cancer. Clin Colorectal Cancer. 2018;17:156‐163. 29486916 10.1016/j.clcc.2018.01.010

[22] Farr KP , Safwat A . Palmar‐plantar erythrodysesthesia associated with chemotherapy and its treatment. Case Rep Oncol. 2011;4:229‐235. 21537373 10.1159/000327767 PMC3085037

[23] Dean LC . Therapy and HLA Genotype In Pratt V, McLeod H, Rubinstein W, et al., eds Medical Genetics Summaries. Bethesda, MD: National Center for Biotechnology Information (US); 2012.

