# Supplementary material for: ToxNav germline genetic testing and PROMinet digital mobile application toxicity monitoring: Results of a prospective single‐center clinical utility study—PRECISE study
Source: Cancer Med. 2019 Sep 4;8(14):6305–14. doi: 10.1002/cam4.2529 (PMC6797583; doi:10.1002/cam4.2529)
Supplement: Supplementary file 1 [file CAM4-8-6305-s001.pdf]

COLOTOX 5FU/CAPECITABINE
TOXICITY MUTATION ANALYSIS

Report Form

PATIENT INFORMATION | SAMPLE INFORMATION

INITIALS:

DOB:

PATIENT ID:

DATE TESTED: 21/01/2019

COLOTOX ASSAY DESCRIPTION

ColoTox assay uses DNA sequencing to determine if a patient has variations in their germline DNA which have been shown to predispose for severe toxicity (CTC Grade 3/4) such as stomatitis, diarrhoea, neutropenia, hand foot syndrome and neurotoxicity upon treatment with a standard dose of 5-fluorouracil or Capecitabine the oral 5-fluorouracil (5-FU) derivative. This toxicity is largely contributed to through dihydropyrimidine dehydrogenase deficiency (DPD) or partial DPD deficiency as listed on the drug labels. The test results will indicate Critical Risk (DPD deficient), High Risk (partial DPD activity) or Standard Risk (normal DPD activity). A list of variants detected is provided and a suggested starting dose modulation. The Risk is calculated using analysis 1046 individuals receiving Capecitabine treatment in the QUASAR2 clinical study[1] and meta-analysis of data from 16 published studies with 4,855 patients receiving 5FU monotherapy or combination therapy[2] [3]. The clinical advice is provided as advised by the Clinical Pharmacogenetics Implementation Consortium guidelines for dihydropyrimidine dehydrogenase genotype and fluoropyrimidine dosing.[4]

RESULTS

**High Risk**	The test indicates this individual has at least 2x the risk of grade 3/4 toxicity using a standard dose of Capecitabine or 5FU monotherapy in comparison to the Standard Risk group. The variants detected are strongly associated with Partial Dihydro Pyrimidine Dehydrogenase (DPD) Deficiency.

CLINICAL INTERPRETATION

For patients identified as HIGH RISK and therefore likely to show PARTIAL DPD DEFICIENCY, with no other contradicting factors, a Capecitabine or 5FU monotherapy dose modulation of 50% is recommended.

ADDITIONAL INFORMATION

Chief Medical Officer: David Kerr CBE FRCP FMedSci

This report is provided by Oxford Cancer Biomarkers Ltd, Magdalen Centre, Oxford Science Park, Robert Robinson Avenue, Oxford, OX44GA, United Kingdom, Tel: +44 (0)1865 784743.Analysis carried out by Source BioScience which is CPA accredited equivalent to ISO15189 and is licensed by the Human Tissue Authority. Source BioScience Healthcare, 1 Orchard Place, Nottingham Business Park, Nottingham, NG8 6PX, United Kingdom, Tel: +44 (0)115 973 9056.

[1] The QUASAR2 study is a phase III randomised controlled trial of adjuvant capecitabine (Xeloda) (1250 mg/m2 twice daily for 14 days every 3 weeks, total of 8 cycles) ± bevacizumab (Avastin) (7.5 mg/kg every 3 weeks) following resection of stage II/III CRC. 1046 patients were selected for genetic study based on availability of clinical data and informed consent.[2] Dan Rosmarin et al A candidate gene study of capecitabine-related toxicity identifies three new DPYD toxicity variants and a putative role for ENOSF1 rather than TYMS, Gut. 2014 Mar 19. doi: 10.1136/gutjnl-2013-306571. PMID: 24647007[3] Dan Rosmarin et al Genetic Markers of Toxicity From Capecitabine and Other Fluorouracil-Based Regimens: Investigation in the QUASAR2 Study, Systematic Review, and Meta-Analysis Dan Rosmarin, J Clinical Oncology Apr 1, 2014:1031-1039. PMID: 24590654[4] Caudle et al. Clinical Pharmacogenetics Implementation Consortium guidelines for dihydropyrimidine dehydrogenase genotype and fluoropyrimidine dosing.Clin Pharmacol Ther. 2013 Dec;94(6):640-5."

COLOTOX 5FU/CAPECITABINE
TOXICITY MUTATION ANALYSIS

Report Form

Oxford Cancer Biomarkers

PATIENT INFORMATION	SAMPLE INFORMATION
INITIALS:	DATE TESTED: 21/01/2019
DOB:	
PATIENT ID:	

VARIANTS DETECTED

rs2612091 A/G, Heterozygote deficiency allele: DYPD rs115232898

VARIANTS TESTED

rs67376798, rs3918290, rs12132152, DPYD A551T, rs2612091 A/G, DPYD 257C>T;Pro86Leu, rs72549309, rs72549308, rs72549307, DPYD 731A>C;Glu244Val, rs1801266, DPYD 1039-1042delTG, DPYD IVS11+1G>T, rs72549304, rs55886062, rs72549303, rs72547601, rs1801268, rs115232898

VARIANTS WITH NO RESULT

DPYD A551T, rs55886062

David Kerr 24th January,2019
