# Supplementary material for: ToxNav germline genetic testing and PROMinet digital mobile application toxicity monitoring: Results of a prospective single‐center clinical utility study—PRECISE study
Source: Cancer Med. 2019 Sep 4;8(14):6305–14. doi: 10.1002/cam4.2529 (PMC6797583; doi:10.1002/cam4.2529)
Supplement: Supplementary file 2 [file CAM4-8-6305-s002.docx]

Supplementary Table 1: Germline variants analysed in the ToxNav test and corresponding frequencies observed in the PRECISE clinical cohort.

| Colotox ID | Gene | Variant | Allele count | Allele freq % | Patient freq % |
| --- | --- | --- | --- | --- | --- |
| Variant1 | DPYD Exon 22 | rs67376798 | 0 | 0.00 | 0.00 |
| Variant2 | DPYD Exon 14 | rs3918290 | 0 | 0.00 | 0.00 |
| Variant3 | DPYD Intronic | rs12132152 | 4 | 3.39 | 6.78 |
| Variant4 | DPYD Intronic | rs7548189 | 15 | 12.71 | 23.73 |
| Variant5 | DPYD Exon 13 | A551T | 1 | 0.85 | 1.69 |
| Variant6 | TYMS/ENOSF1 | rs2612091 | 54 | 45.76 | 66.10 |
| Variant7 | DPYD Exon 4 | 257C>T;Pro86Leu | 0 | 0.00 | 0.00 |
| Variant8 | DPYD Exon 4 | rs72549309 | 0 | 0.00 | 0.00 |
| Variant9 | DPYD Exon 6 | rs72549308 | 0 | 0.00 | 0.00 |
| Variant10 | DPYD Exon 6 | rs72549307 | 0 | 0.00 | 0.00 |
| Variant11 | DPYD Exon 7 | 731A>C;Glu244Val | 0 | 0.00 | 0.00 |
| Variant12 | DPYD Exon 7 | rs1801266 | 0 | 0.00 | 0.00 |
| Variant13 | DPYD Exon 10 | 1039-1042delTG | 0 | 0.00 | 0.00 |
| Variant14 | DPYD Exon 14 | IVS11+1G>T | 0 | 0.00 | 0.00 |
| Variant15 | DPYD Exon 12 | rs72549304 | 0 | 0.00 | 0.00 |
| Variant16 | DPYD Exon 13 | rs55886062 | 0 | 0.00 | 0.00 |
| Variant17 | DPYD Exon 14 | rs72549303 | 0 | 0.00 | 0.00 |
| Variant18 | DPYD Exon 21 | rs1801267 | 0 | 0.00 | 0.00 |
| Variant19 | DPYD Exon 23 | rs72547601 | 0 | 0.00 | 0.00 |
| Variant20 | DPYD Exon 23 | rs1801268 | 0 | 0.00 | 0.00 |
| Variant21 | DPYD Exon 6 | rs115232898 | 0 | 0.00 | 0.00 |
